# 

**Published:** 1879-05

**Authors:** 


					﻿THE BUFFALO
MEDICAL AND SURGICAL
JOURNAL.
VOL. XVIII.	MAY, 1879.	NO. 10.
				

